# Underestimated Prevalence of HIV, Hepatitis B Virus (HBV), and Hepatitis D Virus (HDV) Triple Infection Globally: Systematic Review and Meta-analysis

**DOI:** 10.2196/37016

**Published:** 2022-11-29

**Authors:** Sisi Chen, Feng Ren, Xiaojie Huang, Ling Xu, Yao Gao, Xiangying Zhang, Yaling Cao, Zihao Fan, Yuan Tian, Mei Liu

**Affiliations:** 1 Beijing Institute of Hepatology Beijing Youan Hospital Capital Medical University Beijing China; 2 Department of Oncology Beijing Youan Hospital Capital Medical University Beijing China; 3 Clinical and Research Center for Infectious Diseases Beijing Youan Hospital Capital Medical University Beijing China

**Keywords:** HIV, HBV, HDV, triple infection, epidemiology, public health

## Abstract

**Background:**

Hepatitis delta virus (HDV) is a satellite RNA virus that relies on hepatitis B virus (HBV) for transmission. HIV/HBV/HDV coinfection or triple infection is common and has a worse prognosis than monoinfection.

**Objective:**

We aimed to reveal the epidemiological characteristics of HIV/HBV/HDV triple infection in the global population.

**Methods:**

A systematic literature search in PubMed, Embase, and the Cochrane Library was performed for studies of the prevalence of HIV/HBV/HDV triple infection published from January 1, 1990, to May 31, 2021. The Der Simonian-Laird random effects model was used to calculate the pooled prevalence.

**Results:**

We included 14 studies with 11,852 participants. The pooled triple infection rate in the global population was 7.4% (877/11,852; 95% CI 0.73%-29.59%). The results of the subgroup analysis showed that the prevalence of triple infection was significantly higher in the Asian population (214/986, 21.4%; 95% CI 7.1%-35.8%), in men (212/5579, 3.8%; 95% CI 2.5%-5.2%), and in men who have sex with men (216/2734, 7.9%; 95% CI 4.3%-11.4%). In addition, compared with people living with HIV, the HIV/HBV/HDV triple infection rate was higher in people with hepatitis B.

**Conclusions:**

This meta-analysis suggests that the prevalence of HIV/HBV/HDV triple infection in the global population is underestimated, and we should focus more effort on the prevention and control of HIV/HBV/HDV triple infection.

**Trial Registration:**

PROSPERO CRD42021273949; https://www.crd.york.ac.uk/prospero/display_record.php?RecordID=273949

## Introduction

Hepatitis D virus (HDV) is a peculiar, small, defective virus that requires the assistance of hepatitis B virus (HBV) surface antigen (HBsAg) for replication and pathogenesis [1]. Accordingly, HDV infection can occur via either coinfection with HBV or superinfection in patients with chronic hepatitis B. The main transmission routes of HDV are parenteral and sexual contact. In addition, mother-to-child transmission can occur [2]. Despite being a defective virus, HDV infection is widely perceived as the most severe and aggressive form of human viral hepatitis. Approximately 10% to 15% of patients with hepatitis D progress to cirrhosis within 1 year to 2 years, and 70% to 80% of patients progress to cirrhosis within 5 years to 10 years [3]. Moreover, HDV infection is more prone to hepatic decompensation and is associated with a higher risk of hepatocellular carcinoma [4]. However, HDV infection has been considered a relatively rare disease over the past decades as a result of the universal promotion of HBV vaccination and the clinical neglect of HDV detection. According to recent meta-analyses, the approximate HDV infection rate is 4.5% to 14.57% in the HBsAg-positive population, affecting up to nearly 72 million individuals worldwide [5,6].

Given the shared transmission routes with HIV, HIV/HBV/HDV triple infection is relatively common [7]. HIV/HBV/HDV triple infection is not only widespread but also associated with worse outcomes than monoinfection. First, it can have a negative impact on disease progression for people living with HIV. Combination with hepatitis virus infection may promote immune activation, causing dysfunction of CD4^+^ and CD8^+^ T lymphocytes and natural killer cells, resulting in poor immune recovery after antiretroviral therapy, thus affecting AIDS disease progression [8,9]. Additionally, HIV combined with HBV or HDV infection significantly reduces the clearance rate of these 2 types of hepatitis virus and prolongs the course of hepatitis [10]. Meanwhile, liver fibrosis is significantly accelerated after coinfection, and patients are also at higher risk of mortality due to liver cirrhosis, hepatic decompensation, hepatocellular carcinoma, and other liver diseases [11,12]. Significantly higher rates of poorer prognosis also occur. Thus, the disease burden of HIV/HBV/HDV triple infection appears more serious than initially expected.

There have been several meta-analyses exploring the global HIV/HBV coinfection rate; however, the prevalence of HIV/HBV/HDV triple infection remains largely unknown. Chu et al [13] examined the prevalence of multiple hepatitis viruses and HIV infection among drug users in Taiwan and found that HIV/HBV/HDV infection rates were as high as 16.7% among HIV-positive drug users. Shen et al [14] pooled the HIV/HBV/HDV triple infection rate in the global population from 2002 to 2018 and estimated a triple infection rate of only 1.03% in people living with HIV. In addition, Nicolini et al [15], in 2015, tested triple infection in blood samples from the Italian general population and found a triple infection rate of 3.5%. In general, the results of current studies on HIV/HBV/HDV triple infection rates fluctuate widely, and some studies may underestimate triple infection rates to some extent due to the small number of included samples or limitations of the included population characteristics [14]. This systematic review and meta-analysis aimed to determine a high reliability estimate of the prevalence of HIV/HBV/HDV triple infection in people with HBV infection or living with HIV globally.

## Methods

This meta-analysis was conducted based on the Preferred Reporting Items for Systematic Reviews and Meta-Analysis (PRISMA) guidelines, and it was registered in PROSPERO (CRD42021273949).

### Search Strategy

A systematic literature search in PubMed, Embase, and the Cochrane Library was performed for studies on the prevalence of HBV, HIV/AIDS, and HDV triple infection published from January 1, 1990, to May 31, 2021.

### Selection Criteria

#### Inclusion Criteria

Studies were selected based on the following inclusion criteria: (1) study participants were HIV or HBV monoinfected or HBV and HIV coinfected; (2) the diagnosis of infection with HIV or HBV met the international uniform standards; (3) studies related to the prevalence of triple infection were cross-sectional studies, and those related to incidence and risk factors associated with triple infection were case control studies or cohort studies; (4) in the original study, coinfection with HBV was defined as HBsAg-positive, coinfection with HDV was defined as anti-HDV positive, and coinfection with HIV was confirmed by Western blot.

#### Exclusion Criteria

Studies were excluded if they (1) were case reports or review articles, (2) had a research sample size of less than 50 participants, (3) were duplicate studies, or (4) had incomplete or unclear study information. The study screening was carried out independently by 2 reviewers, who both read the full text and screened the studies that met the inclusion and exclusion criteria. Disagreements between reviewers about inclusion were resolved by consulting third-party experts.

### Data Extraction

Two researchers independently extracted and coded data using an Excel spreadsheet. The data obtained included basic information of the included studies, including the first author, year of publication, study period, research type, study location, age or sex distribution, total number of participants, number of participants with HIV/HBV/HDV triple infection, and crude prevalence rate.

### Quality Assessment

The Newcastle-Ottawa Scale (NOS; 11 items in total, out of 11 points) [16] was used to evaluate the quality of the included cohort studies, and the Agency for Healthcare Research and Quality (AHRQ) questionnaire (9 items in total, out of 10 points) [17] was used to evaluate the quality of the included cross-sectional studies. We used 3 grades: A, B, and C. Grade A corresponds to 7-10 points on the NOS scale and 8-11 “Yes” responses on the AHRQ questionnaire. Grade B corresponds to 3-6 points on the NOS scale and 4-7 “Yes” responses on the AHRQ questionnaire. Grade C corresponds to 0-2 points on the NOS scale and 0-3 “Yes” responses on the AHRQ questionnaire.

### Statistical Analysis

We used Stata software for the meta-analysis. Heterogeneity was assessed statistically using the *I*^2^ measurement. The threshold for the heterogeneity test result was 0.05, and that of the level of goodness-of-fit test was 0.10. If heterogeneity was high (*P*<.10 or *I*^2^>50%), the random effects model was used to calculate pooled prevalence estimates with 95% CIs. Subgroup analysis was conducted according to the basic disease, research continent, research country, and HIV transmission route to explore the source of heterogeneity. The Egger linear regression method combined with the observation funnel plot was used to evaluate the publication bias. We evaluated the stability of the model through a sensitivity analysis.

## Results

### Literature Search

**Figure 1:**
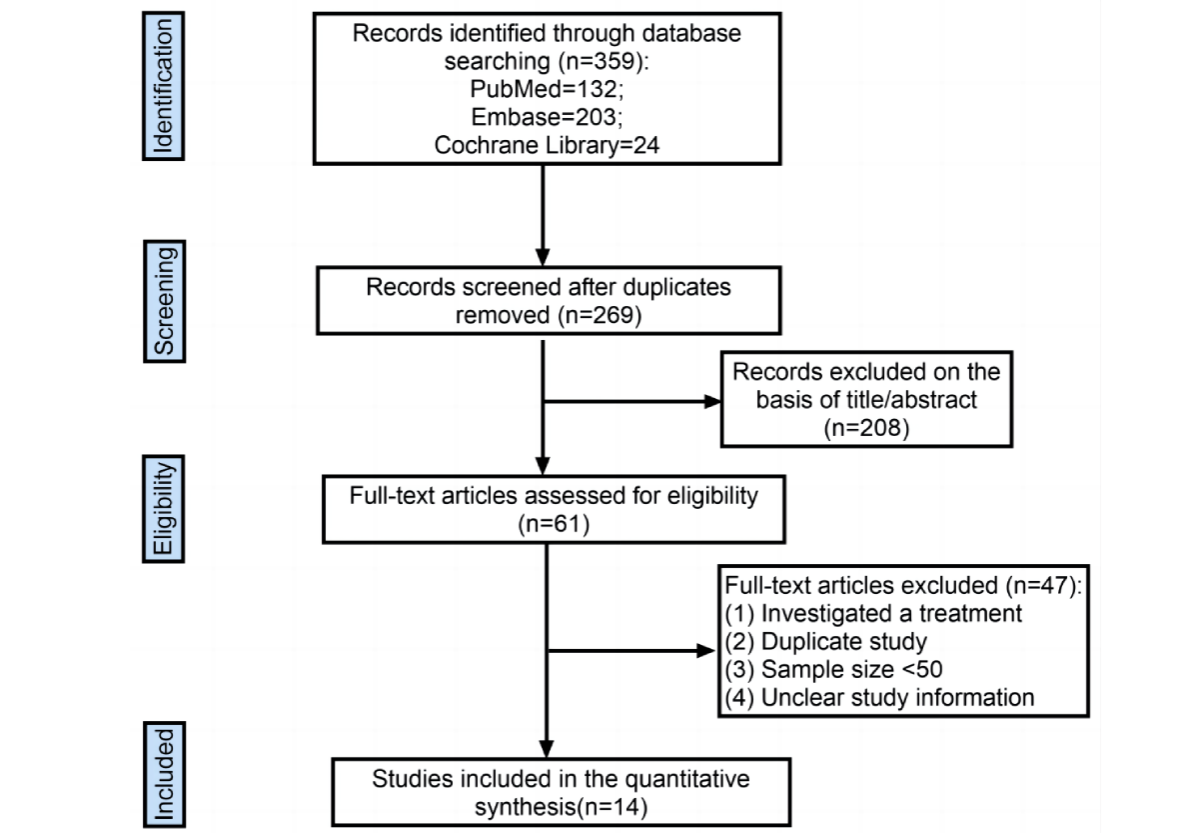
Literature identification process.

The detailed flow of the literature search is shown in Figure 1. The literature search yielded 359 studies from 3 databases (Embase: 203; PubMed: 132; Cochrane Library: 24). After removing 90 duplicates, the remaining literature was screened for titles and abstracts. Of the 61 studies that underwent full-text assessment, we excluded 48 studies because they investigated treatments, were duplicate studies, had a small sample size (<50), or had unclear study information. Finally, 14 articles with a total of 11,852 participants were included [10,11,15,18-28]. Of the included studies, 9 were prospective cohort studies, and 5 were cross-sectional studies (Table 1).

**Table 1:** Main characteristics of the included studies assessing the prevalence of triple infection.

Study number	First author	Type of study	Sample size, n	Male participants, n	Study population	HBV^a^/HDV^b^/HIV triple infection		Year		Continent		Country		Quality evaluation
						Sample size, n	Prevalence, %							
1	Soriano [18]	Cohort	5342	NR^c^	HIV	61	1.14	2011	Europe		NR		B^d^	
2	Hønge [19]	Cross-sectional	576	180	HIV	18	3.13	2014	Africa		Guinea-Bissau		A^e^	
3	Dény [20]	Cross-sectional	206	NR	HIV	19	9.22	1993	Europe		France		B	
4	Coffie [21]	Cross-sectional	791	319	HIV	10	1.26	2017	Africa		NR		A	
5	Ifeorah [22]	Cohort	1102	450	HIV	8	0.73	2017	Africa		Nigeria		A	
6	Nicolini [15]	Cross-sectional	454	NR	HBV	16	3.52	2015	Europe		Italy		B	
7	Saravanan [23]	Cohort	450	270	HBV	4	0.89	2015	Europe		Italy		A	
8	Butler [24]	Cohort	1928	806	HBV	390	20.23	2018	Africa		Cameroon		A	
9	Chang [25]	Cross-sectional	507	NR	HBV	150	29.59	2011	Asia		China (Taiwan)		B	
10	Oprea [26]	Cohort	205	NR	HIV+HBV	21	10.24	2009	Europe		Romania		B	
11	Béguelin [10]	Cohort	771	NR	HIV+HBV	117	15.18	2016	Europe		Switzerland		A	
12	Sheng [11]	Cohort	104	100	HIV+HBV	26	25.00	2006	Asia		China (Taiwan)		A	
13	Lee [27]	Cohort	375	363	HIV+HBV	38	10.13	2014	Asia		China (Taiwan)		B	
14	Boyd [28]	Cohort	308	259	HIV+ HBV	12	3.90	2009	Europe		France		A	

^a^HBV: hepatitis B virus.

^b^HDV: hepatitis D virus.

^c^NR: not reported.

^d^Grade B corresponds to 3-6 points on the Newcastle-Ottawa Scale (NOS) scale and 4-7 “Yes” responses on the Agency for Healthcare Research and Quality (AHRQ) questionnaire.

^e^Grade A corresponds to 7-10 points on the NOS scale and 8-11 “Yes” responses on the AHRQ questionnaire.

### Study Characteristics

The detailed characteristics of the 14 included studies are listed in Table 1. Among the 14 studies, 3 were conducted in Taiwan (China); 2 were conducted in France; 2 were conducted in Italy; 1 each was conducted in Guinea-Bissau, Nigeria, Cameroon, and Switzerland; and there were 2 multinational studies, 1 in Europe and 1 in Africa. The sample size ranged from 104 to 5342 participants, with a total sample size across the included studies of 11,852 participants. The prevalence of HIV/HBV/HDV triple infection was evaluated in 4 studies with patients with chronic hepatitis B, 5 studies with people living with HIV, and 5 studies with patients with HIV and HBV coinfection. Quality evaluations were either A or B. The demographic characteristics of the included studies are listed in Table 2. Participants ranged in age from 12 years to 61 years.

**Table 2:** Demographic characteristics of the included studies assessing the prevalence of triple infection.

Study number	First author	Sample size, n	Study population	HBV^a^/HDV^b^/HIV triple infection			
				Sample size, n	Prevalence, %	Age, mean (range)	Male participants, n (%)
1	Soriano [18]	5342	HIV	61	1.14	34 (NR)	44 (72.1)
2	Hønge [19]	576	HIV	18	3.13	NR	NR
3	Dény [20]	206	HIV	19	9.22	NR	NR
4	Coffie [21]	791	HIV	10	1.26	NR	NR
5	Ifeorah [22]	1102	HIV	8	0.73	NR (31-40)	5 (62.5)
6	Nicolini [15]	454	HBV	16	3.52	34.25 (6.16)^d^	14 (87.5)
7	Saravanan [23]	450	HBV	4	0.89	NR (21-40)	4 (100)
8	Butler [24]	1928	HBV	390	20.23	NR	NR
9	Chang [25]	507	HBV	150	29.59	NR	NR
10	Oprea [26]	205	HIV+HBV	21	10.24	16 (12-20)	NR
11	Béguelin [10]	771	HIV+HBV	117	15.18	34 (29-37)	92 (79)
12	Sheng [11]	104	HIV+HBV	26	25.00	35 (25-61)	25 (96.2)
13	Lee [27]	375	HIV+HBV	38	10.13	38 (NR)	36 (94.7)
14	Boyd [28]	308	HIV+HBV	12	3.90	35.2 (NR)	8 (66.7)

^a^HBV: hepatitis B virus.

^b^HDV: hepatitis D virus.

^c^NR: not reported.

^d^Median (SD).

### Meta-analyses of the Data

**Figure 2:**
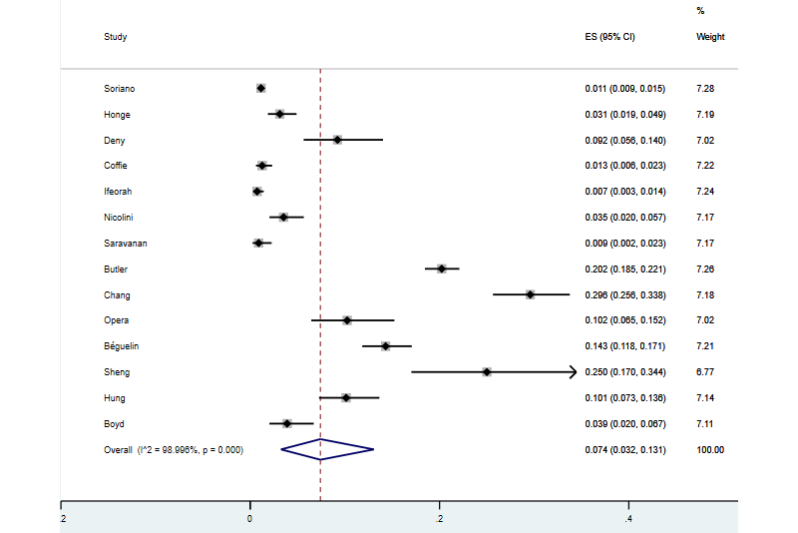
Forest plot showing the prevalence of hepatitis B virus (HBV), hepatitis D virus (HDV), and HIV triple infection in the included studies. ES: effect size.

**Figure 3:**
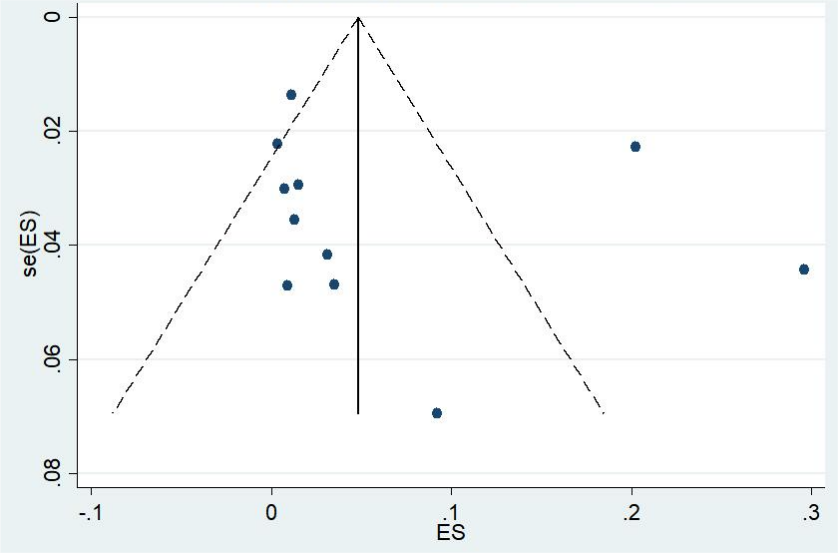
Funnel plot with 95% pseudo confidence limits for all included studies. ES: effect size.

The heterogeneity between studies in the meta-analysis of HIV/HBV/HDV triple infection rate was significant (*I*^2^=98.995%, *P*<.01), and a random effects model was selected to combine the results of the included studies, which showed that the triple infection rate in the global population was 7.4% (877/11,852; 95% CI 0.73%-29.59%; Figure 2). Funnel plots and the Egger test were performed to detect publication bias. The funnel plot was basically symmetrical, and the Egger test results showed no significant statistical evidence of publication bias (*t*=1.13, *P*=.28; Figure 3). In view of the significant heterogeneity of HIV/HBV/HDV triple infection rates in different regions, countries, sexes, ages, sample sizes, basic diseases, and different transmission routes, subgroup analyses were conducted for these factors (Table 3). There was high heterogeneity among the results for all population groups.

**Table 3:** Prevalence of HIV, hepatitis B virus (HBV), and hepatitis D virus (HDV) triple infection in different subgroups.

Subgroup		Studies, n	Triple infection, n	Prevalence, % (95% CI)	*I*^2^, %	*P* value
Global		14	877	7.4 (3.2-13.1)	99.0	<.001
**World region**						
	Europe	7	237	5.7 (3.2-8.2)	95.9	<.001
	Africa	4	426	6.3 (0.7-11.8)	99.3	<.001
	Asia	3	214	21.4 (7.1-35.8)	96.7	<.001
**Country or area**						
	France	2	31	6.3 (1.1-11.5)	81.4	.02
	Italy	2	20	2.1 (0.5-4.7)	86.4	.007
	Cameroon	1	390	20.2 (18.4-22.0)	—^a^	<.001
	Romania	1	21	10.2 (6.1-14.4)	—	—
	Switzerland	1	117	14.3 (11.8-16.9)	—	—
	China (Taiwan)	3	214	21.4 (7.1-35.8)	96.7	<.001
	Guinea-Bissau	1	18	3.1 (1.7-4.5)	—	—
	Nigeria	1	8	0.7 (0.2-1.2)	—	—
**Sex**						
	Male	8	212	3.8 (2.5-5.2)	95.3	<.001
	Female	7	57	0.7 (0.2-1.2)	78.0	<.001
**Age (years)**						
	<30	1	21	5.6 (3.6-7.3)	—	—
	≥30	8	269	5.1 (3.3-6.8)	96.2	<.001
**Sample size**						
	50-500	7	136	7.7 (4.2-11.1)	93.5	<.001
	500-1000	4	282	11.8 (4.0-19.7)	98.9	<.001
	>1000	3	459	7.2 (2.4-11.9)	99.5	<.001
**Study population**						
	HIV	5	116	1.7 (0.9-2.5)	84.8	<.001
	HBV	4	560	13.4 (2.5-24.4)	99.4	<.001
	HIV+HBV	5	201	11.9 (6.5-17.2)	92.5	<.001
**Possible mode of HIV transmission**						
	Injection drug use	6	274	7.4 (2.9-11.9)	97.9	<.001
	Men who have sex with men	6	216	7.9 (4.3-11.4)	98.1	<.001
	Heterosexual transmission	6	201	6.5 (3.4-9.6)	98	<.001
	Other	6	248	10.3 (6.0-14.7)	98.4	<.001

^a^Not available.

We analyzed 14 studies by regional subgroup and found that Asia had the highest pooled prevalence of triple infection at 21.4% (214/986; 95% CI 7.1%-35.8%, *I*^2^=96.7%), and Europe had the lowest prevalence at 5.7% (237/4158; 95% CI 3.2%-8.2%, *I*^2^=95.9%; Table 3, Multimedia Appendix 1). In addition, a subgroup analysis of the prevalence of triple infection according to country was performed, and a total of 12 studies were included, showing that the prevalence in Taiwan (China) was 21.4% (214/986; 95% CI 7.1%-35.8%, *I*^2^=96.7%), the highest prevalence among the 8 countries included. Nigeria had the lowest prevalence at 0.7% (8/1143; 95% CI 0.2%-1.2%). The prevalence rates in the other 6 countries are shown in Table 3 and Multimedia Appendix 2.

We also performed a subgroup analysis by sex and found that men had a higher prevalence than women (228/8862, 3.8%; 95% CI 2.5%-5.2% vs 57/8412, 0.7%; 95% CI 0.3%-1.2%; Table 3, Multimedia Appendix 3). The triple infection rate was 5.6% (21/375; 95% CI 3.6%-7.3%) in those aged <30 years and 5.1% (269/5275; 95% CI 3.3%-6.8%) in those aged ≥30 years (Table 3, Multimedia Appendix 4). In studies with a sample size of 500-1000, the triple infection rate was the highest, at 11.8% (282/2390; 95% CI 4.0%-19.7%; Table 3, Multimedia Appendix 5).

Moreover, population-specific subgroup analyses were performed depending on the characteristics of the populations included in the study, and the results showed that the prevalence of triple infection varied greatly among the different populations. The prevalences of HIV/HBV/HDV triple infection were 13.4% (560/4079; 95% CI 2.5%-24.4%) in patients with chronic hepatitis B, 1.7% (116/6224; 95% CI 0.9%-2.5%) in people living with HIV, and 11.9% (201/1549; 95% CI 6.5%-17.2%) in people with HBV/HIV coinfection (Table 3, Multimedia Appendix 6). Meanwhile, since there are multiple transmission routes for HIV, such as transmission via men who have sex with men, heterosexual transmission, and transmission via injection drug use (IDU), a subgroup analysis of the transmission routes was performed. The pooled prevalence of triple infection was 7.9% (216/2734; 95% CI 4.3%-11.4%) in men who have sex with men, 6.5% (201/3092; 95% CI 3.4%-9.6%) in those with heterosexual transmission, and 7.4% (274/3703; 95% CI 2.9%-11.9%) in those with IDU (Table 3, Multimedia Appendix 7). “Other” modes of transmission include perinatal, risk not identified, and blood transfusion. In addition, given that people with IDU may be more prone to triple infections, we collapsed the data on drug-using people included in the literature and calculated a prevalence of triple infection of 20.6% among people with IDU, which is significantly higher than the prevalence in the total population (Multimedia Appendix 8).

## Discussion

Since the first discovery of the HDV in 1977 [29], it is estimated that 15 million to 20 million people have been infected worldwide [30,31]. Given that HDV is a defective virus dependent on the envelope proteins of HBV for assembly and release of infectious virus particles, HDV infection occurs either with or secondary to HBV infection. People infected with HDV can also have other viral infections, and chronic HDV infection is considered to be the most severe form of viral hepatitis infection in humans [32]. HIV was first reported in 1981, and the number of people living with HIV and deaths due to illness have remained high for a long time [33]. The virus is widely prevalent worldwide. As of 2018, there were more than 37.9 million people living with HIV in the world, and a total of 35 million people have died from AIDS-related diseases [34]. HIV infection causes progressive immunodeficiency, making people living with HIV highly susceptible to coinfection with other diseases. HBV and HDV are common viruses for coinfection in people living with HIV, as all 3 share the same route of infection [14,35]. Studies have shown that HIV coinfection with HBV and HDV is widespread in various regions of the world, but the coinfection rate varies among countries and regions [36]. HIV coinfection with HBV or HDV can cause more serious damage to the body than a single infection, and the harm of triple infection is even more serious. Therefore, it is of great public health significance to actively prevent such coinfections [37,38].

The results of this meta-analysis showed that the prevalence of HIV/HBV/HDV triple infection was 7.4%. The results of the subgroup analysis showed significant regional differences in global triple infection rates. The prevalence of triple infection is significantly higher in Asia, especially in Taiwan and China, than in other countries or regions. These results may be caused by several reasons. First, the included studies included people with IDU; IDU is a high-risk factor that may promote triple infections [39]. In addition, the higher prevalence rate might be due to the small sample size of the included studies, which may not accurately reflect the real situation of triple infection in this population. We found that the characteristics of the study population greatly influenced the prevalence; for example, the prevalence of triple infection in the Cameroon region was higher because the study population was HBsAg-positive and in a general hospital, which itself confers high risk for infection. Among the different sexes, the results of this meta-analysis showed a higher rate of triple infection in men than in women, which is consistent with several other studies [40,41]. This review suggested that men with HIV or HBV infection may have a higher prevalence of triple infection, and more attention should be given to the prognosis of their triple infection.

There were significant differences in the rate of triple infection for people living with HIV from different population sources, which was similar to rates for HIV/HBV coinfection [42]; however, for triple infection, men who have sex with men and people living with HIV are particularly worthy of attention. The rate of triple coinfection in this population reached 7.9%, exceeding the rate of triple infection in other populations. Nevertheless, the literature related to these special populations suffers from the same shortcomings as aforementioned, with small numbers, regional limitations, small sample sizes, and mostly poor quality, which urgently needs to be supplemented with similar studies to help understand the current situation.

Furthermore, through the analysis of the results, we found a very interesting phenomenon. The rate of HIV/HBV/HDV triple infection was higher in people with HBV monoinfection than in people with HIV monoinfection or people with HBV/HIV coinfection. However, the credibility and reason for these findings are still unclear, and a large number of clinical studies is needed to better confirm the results.

There were several limitations in this systematic review. Although strict inclusion and exclusion criteria were established and the quality of the included literature was evaluated using the NOS or AHRQ statement entries during the search and screening processes, there was still some subjectivity in the evaluation of the literature due to the lack of accepted quality evaluation criteria, which may lead to some selection bias in the included literature. The results of the sensitivity analysis in this systematic review showed that there was a certain selection bias. In addition, the wide inclusion criteria in this study produced significant heterogeneity that could not be explained. We used a random effects model with subgroup analyses whenever possible to reduce the effect of heterogeneity. Furthermore, the population included in this meta-analysis included people with HBV infection or HIV, lacking a comparable general population; some of these patients were drug users, which would increase the overall prevalence to some extent; and the evidence base had some shortcomings.

In summary, the prevalence of HIV/HBV/HDV triple infection in the global population is underestimated. Therefore, during the management and antiviral treatment of patients with HBV/HIV single infection or coinfection, they should be screened for HIV/HBV/HDV triple infection in a timely manner. In addition, the prevention and treatment of coinfection should be combined with antiviral treatment to provide comprehensive prevention and treatment of triple infection and improve the quality of survival for this population. Additionally, the rates of triple infection in the two special groups of men who have sex with men and people with IDU are also worthy of attention. However, because there are few relevant studies, it is impossible to accurately evaluate the current status of rates of triple infection in the global populations of men who have sex with men and people with IDU [43,44]. More research is urgently needed to provide evidence, identify high-risk populations, and guide the formulation and improvement of prevention and control strategies for HIV/HBV/HDV infection.

## Appendix

Multimedia Appendix 1 Forest plot showing the meta-analysis of triple infection in different regions.

Multimedia Appendix 2 Forest plot showing meta-analysis of triple infection in different countries.

Multimedia Appendix 3 Forest plot showing the meta-analysis of triple infection in different genders.

Multimedia Appendix 4 Forest plot showing the meta-analysis of triple infection for different ages.

Multimedia Appendix 5 Forest plot showing the meta-analysis of triple infection in different sample sizes.

Multimedia Appendix 6 Forest plot showing the meta-analysis of triple infection in different study populations.

Multimedia Appendix 7 Forest plot showing the meta-analysis of triple infection for different HIV transmission routes.

Multimedia Appendix 8 Forest plot showing the meta-analysis of triple infection in people who inject drugs.

## Abbreviations

AHRQ: Agency for Healthcare Research and Quality
HBsAg: hepatitis B virus surface antigen
HBV: hepatitis B virus
HDV: hepatitis D virus
IDU: injection drug use
NOS: Newcastle-Ottawa Scale

## References

[1] Taylor JM (2020). Infection by hepatitis delta virus. Viruses.

[2] Lanini S, Ustianowski A, Pisapia R, Zumla A, Ippolito G (2019). Viral hepatitis: etiology, epidemiology, transmission, diagnostics, treatment, and prevention. Infect Dis Clin North Am.

[3] Miao Z, Zhang S, Ou X, Li S, Ma Z, Wang W, Peppelenbosch MP, Liu J, Pan Q (2020). Estimating the global prevalence, disease progression, and clinical outcome of hepatitis delta virus infection. J Infect Dis.

[4] Zhang Z, Urban S (2021). New insights into HDV persistence: The role of interferon response and implications for upcoming novel therapies. J Hepatol.

[5] Chen H, Shen D, Ji D, Han P, Zhang W, Ma J, Chen W, Goyal H, Pan S, Xu H (2019). Prevalence and burden of hepatitis D virus infection in the global population: a systematic review and meta-analysis. Gut.

[6] Stockdale AJ, Kreuels B, Henrion MYR, Giorgi E, Kyomuhangi I, de Martel C, Hutin Y, Geretti AM (2020). The global prevalence of hepatitis D virus infection: Systematic review and meta-analysis. J Hepatol.

[7] Soriano V, Sherman KE, Barreiro P (2017). Hepatitis delta and HIV infection. AIDS.

[8] Nisini R, Paroli M, Accapezzato D, Bonino F, Rosina F, Santantonio T, Sallusto F, Amoroso A, Houghton M, Barnaba V (1997). Human CD4+ T-cell response to hepatitis delta virus: identification of multiple epitopes and characterization of T-helper cytokine profiles. J Virol.

[9] Ferrante ND, Lo Re V (2020). Epidemiology, natural history, and treatment of hepatitis delta virus infection in HIV/hepatitis B virus coinfection. Curr HIV/AIDS Rep.

[10] Béguelin C, Moradpour D, Sahli R, Suter-Riniker F, Lüthi A, Cavassini M, Günthard HF, Battegay M, Bernasconi E, Schmid P, Calmy A, Braun DL, Furrer H, Rauch A, Wandeler G, Swiss HIV Cohort Study (2017). Hepatitis delta-associated mortality in HIV/HBV-coinfected patients. J Hepatol.

[11] Sheng WH, Hung CC, Kao JH, Chang SY, Chen MY, Hsieh SM, Chen PJ, Chang SC (2007). Impact of hepatitis D virus infection on the long-term outcomes of patients with hepatitis B virus and HIV coinfection in the era of highly active antiretroviral therapy: a matched cohort study. Clin Infect Dis.

[12] Fernández-Montero JV, Vispo E, Barreiro P, Sierra-Enguita R, de Mendoza C, Labarga P, Soriano V (2014). Hepatitis delta is a major determinant of liver decompensation events and death in HIV-infected patients. Clin Infect Dis.

[13] Chu F, Chiang S, Su F, Chang Y, Cheng S (2009). Prevalence of human immunodeficiency virus and its association with hepatitis B, C, and D virus infections among incarcerated male substance abusers in Taiwan. J Med Virol.

[14] Shen D, Han P, Ji D, Chen H, Cao W, Goyal H, Xu H (2021). Epidemiology estimates of hepatitis D in individuals co-infected with human immunodeficiency virus and hepatitis B virus, 2002-2018: A systematic review and meta-analysis. J Viral Hepat.

[15] Nicolini LA, Taramasso L, Schiavetti I, Giannini EG, Beltrame A, Feasi M, Cassola G, Grasso A, Bartolacci V, Sticchi L, Picciotto A, Viscoli C, Ligurian HBV Study Group (2015). Epidemiological and clinical features of hepatitis delta in HBsAg-positive patients by HIV status. Antivir Ther.

[16] Stang A (2010). Critical evaluation of the Newcastle-Ottawa scale for the assessment of the quality of nonrandomized studies in meta-analyses. Eur J Epidemiol.

[17] Farquhar M, Hughes RG (2008). AHRQ Quality Indicators. Patient Safety and Quality: An Evidence-Based Handbook for Nurses.

[18] Soriano V, Grint D, d'Arminio Monforte A, Horban A, Leen C, Poveda E, Antunes F, de Wit S, Lundgren J, Rockstroh J, Peters L (2011). Hepatitis delta in HIV-infected individuals in Europe. AIDS.

[19] Hønge BL, Jespersen S, Medina C, Té DS, da Silva ZJ, Lewin S, Østergaard L, Erikstrup C, Wejse C, Laursen AL, Krarup H, Bissau HIV cohort study group (2014). Hepatitis B and delta virus are prevalent but often subclinical co-infections among HIV infected patients in Guinea-Bissau, West Africa: a cross-sectional study. PLoS One.

[20] Dény P, Lecot C, Jeantils V, Ovaguimian L, Krivitzky A, Bréchot C (1993). Polymerase chain reaction-based detection of hepatitis D virus genome in patients infected with human immunodeficiency virus. J Med Virol.

[21] Coffie PA, Tchounga BK, Bado G, Kabran M, Minta DK, Wandeler G, Gottlieb GS, Dabis F, Eholie SP, Ekouevi DK (2017). Prevalence of hepatitis B and delta according to HIV-type: a multi-country cross-sectional survey in West Africa. BMC Infect Dis.

[22] Ifeorah IM, Bakarey AS, Adeniji JA, Onyemelukwe FN (2017). Seroprevalence of hepatitis B and delta viruses among HIV-infected population attending anti-retroviral clinic in selected health facilities in Abuja, Nigeria. J Immunoassay Immunochem.

[23] Saravanan S, Madhavan V, Velu V, Murugavel KG, Waldrop G, Solomon SS, Balakrishnan P, Kumarasamy N, Smith DM, Mayer KH, Solomon S, Thyagarajan SP (2014). High prevalence of hepatitis delta virus among patients with chronic hepatitis B virus infection and HIV-1 in an intermediate hepatitis B virus endemic region. J Int Assoc Provid AIDS Care.

[24] Butler EK, Rodgers MA, Coller KE, Barnaby D, Krilich E, Olivo A, Cassidy M, Mbanya D, Kaptue L, Ndembi N, Cloherty G (2018). High prevalence of hepatitis delta virus in Cameroon. Sci Rep.

[25] Chang S, Yang C, Ko W, Liu W, Lin C, Wu C, Su Y, Chang S, Chen M, Sheng W, Hung C, Chang S (2011). Molecular epidemiology of hepatitis D virus infection among injecting drug users with and without human immunodeficiency virus infection in Taiwan. J Clin Microbiol.

[26] Oprea C., Radoi R., Ungureanu E., Tardei G., Ene L., Erhan R., Tetradov S., Ionescu C., Ceausu E., Duiculescu D. (2010). Hepatitis delta in HIV-1-infected Romanian adolescents. HIV Med.

[27] Lee CY, Tsai HC, Lee SS, Wu KS, Sy CL, Chen JK, Chen YS (2015). Higher rate of hepatitis events in patients with human immunodeficiency virus, hepatitis B, and hepatitis D genotype II infection: a cohort study in a medical center in southern Taiwan. J Microbiol Immunol Infect.

[28] Boyd A, Lacombe K, Miailhes P, Gozlan J, Bonnard P, Molina J, Lascoux-Combe C, Serfaty L, Gault E, Desvarieux M, Girard P (2010). Longitudinal evaluation of viral interactions in treated HIV-hepatitis B co-infected patients with additional hepatitis C and D virus. J Viral Hepat.

[29] Toy M, Ahishali E, Yurdaydın C (2020). Hepatitis delta virus epidemiology in the industrialized world. AIDS Rev.

[30] Koh C, Heller T, Glenn JS (2019). Pathogenesis of and new therapies for hepatitis D. Gastroenterology.

[31] Mentha N, Clément S, Negro F, Alfaiate D (2019). A review on hepatitis D: from virology to new therapies. J Adv Res.

[32] Tseligka ED, Clément S, Negro F (2021). HDV pathogenesis: unravelling Ariadne's thread. Viruses.

[33] Eisinger RW, Fauci AS (2018). Ending the HIV/AIDS pandemic. Emerg Infect Dis.

[34] Ghosn J, Taiwo B, Seedat S, Autran B, Katlama C (2018). HIV. Lancet.

[35] Singh KP, Crane M, Audsley J, Avihingsanon A, Sasadeusz J, Lewin SR (2017). HIV-hepatitis B virus coinfection: epidemiology, pathogenesis, and treatment. AIDS.

[36] Torimiro JN, Nanfack A, Takang W, Keou CK, Joyce AN, Njefi K, Agyingi K, Domkam I, Takou D, Moudourou S, Sosso S, Mbu RE (2018). Rates of HBV, HCV, HDV and HIV type 1 among pregnant women and HIV type 1 drug resistance-associated mutations in breastfeeding women on antiretroviral therapy. BMC Pregnancy Childbirth.

[37] Assih M, Ouattara AK, Diarra B, Yonli AT, Compaore TR, Obiri-Yeboah D, Djigma FW, Karou S, Simpore J (2018). Genetic diversity of hepatitis viruses in West-African countries from 1996 to 2018. World J Hepatol.

[38] Hu J, Liu K, Luo J (2019). HIV-HBV and HIV-HCV coinfection and liver cancer development. Cancer Treat Res.

[39] Safaie P, Razeghi S, Rouster SD, Privitera I, Sherman KE (2018). Hepatitis D diagnostics: utilization and testing in the United States. Virus Res.

[40] Ziaee M, Sharifzadeh G, Namaee MH, Fereidouni M (2014). Prevalence of HIV and hepatitis B, C, D infections and their associated risk factors among prisoners in Southern Khorasan Province, Iran. Iran J Public Health.

[41] Ramezan Ghorbani N, Qorbani M, Djalalinia S, Kazemzadeh Atoofi M, Tajbakhsh R, Mansourian M, Gorabi AM, Asayesh H, Soleimani A, Noroozi M (2019). Oncogenic viral infections among Iranian hemodialysis patients: a systematic review. Int J Prev Med.

[42] Soares CC, Georg I, Lampe E, Lewis L, Morgado MG, Nicol AF, Pinho AA, Salles RCS, Teixeira SLM, Vicente ACP, Viscidi RP, Gomes SA (2014). HIV-1, HBV, HCV, HTLV, HPV-16/18, and Treponema pallidum infections in a sample of Brazilian men who have sex with men. PLoS One.

[43] Kibaya RM, Lihana RW, Kiptoo M, Songok EM, Ng'ang'a Z, Osman S, Ishizaki A, Bi X, Okoth FA, Ichimura H, Lwembe RM (2015). Characterization of HBV among HBV/HIV-1 co-infected injecting drug users from Mombasa, Kenya. Curr HIV Res.

[44] Sadio AJ, Gbeasor-Komlanvi FA, Konu YR, Sewu EK, Zida-Compaore W, Salou M, Kariyiare BG, Blatome TJ, Dagnra AY, Ekouevi DK (2019). Prevalence of HIV infection and hepatitis B and factors associated with them among men who had sex with men in Togo in 2017. Med Sante Trop.

