# Supplementary figures and images for: Underestimated Prevalence of HIV, Hepatitis B Virus (HBV), and Hepatitis D Virus (HDV) Triple Infection Globally: Systematic Review and Meta-analysis
Source: JMIR Public Health Surveill. 2022 Nov 29;8(11):e37016. doi: 10.2196/37016 (PMC9748799; doi:10.2196/37016)

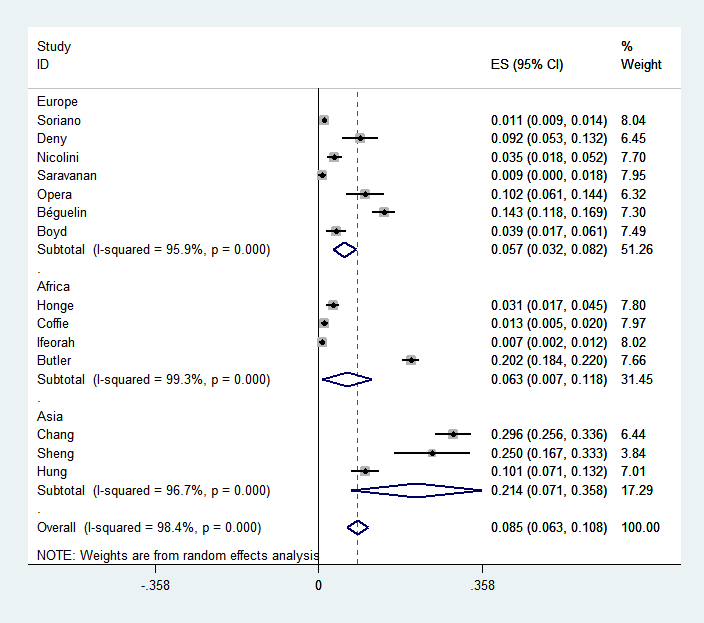

Supplement: Multimedia Appendix 1 [file publichealth_v8i11e37016_app1.png]

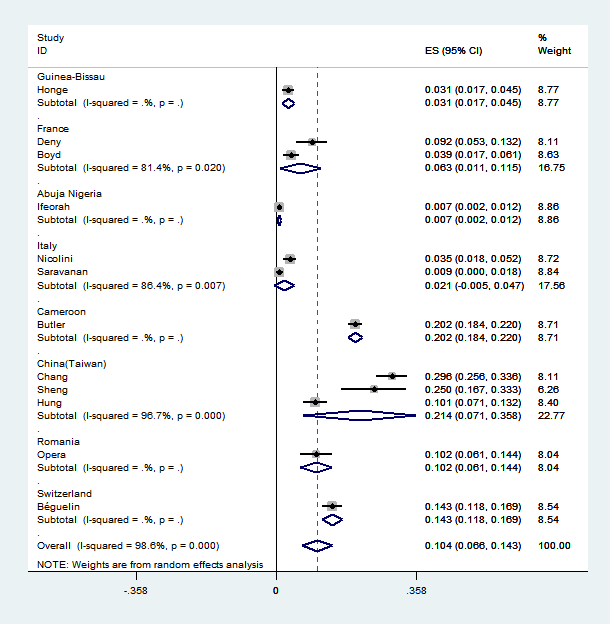

Supplement: Multimedia Appendix 2 [file publichealth_v8i11e37016_app2.png]

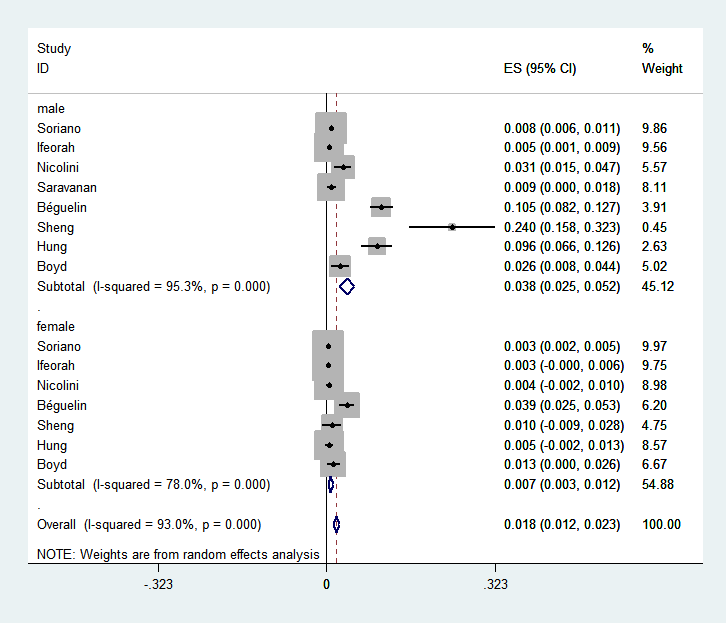

Supplement: Multimedia Appendix 3 [file publichealth_v8i11e37016_app3.png]

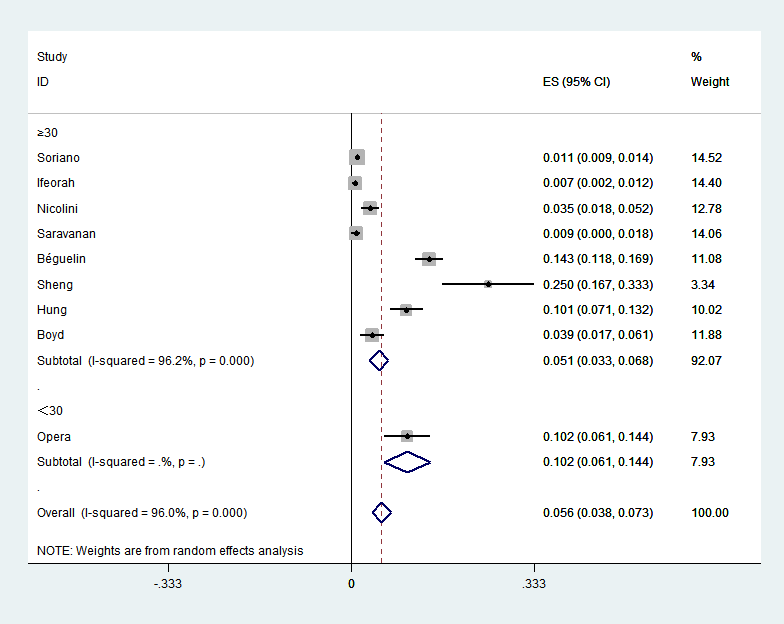

Supplement: Multimedia Appendix 4 [file publichealth_v8i11e37016_app4.png]

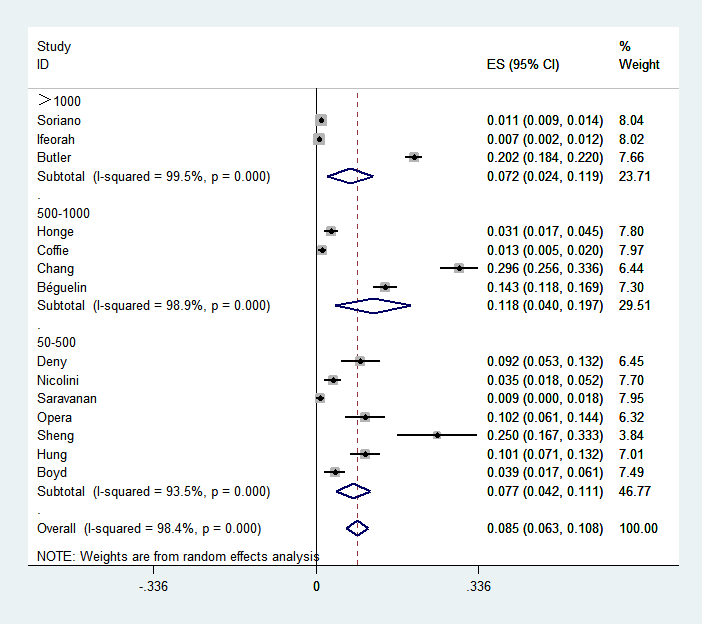

Supplement: Multimedia Appendix 5 [file publichealth_v8i11e37016_app5.png]

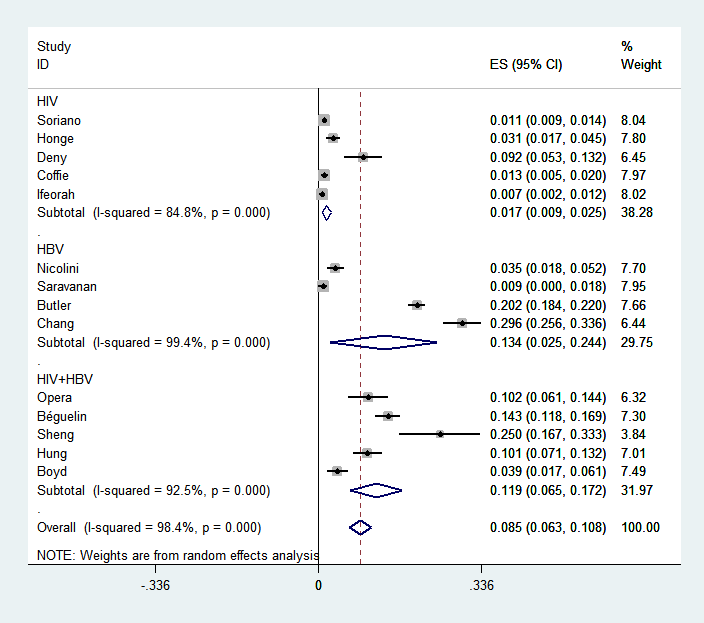

Supplement: Multimedia Appendix 6 [file publichealth_v8i11e37016_app6.png]

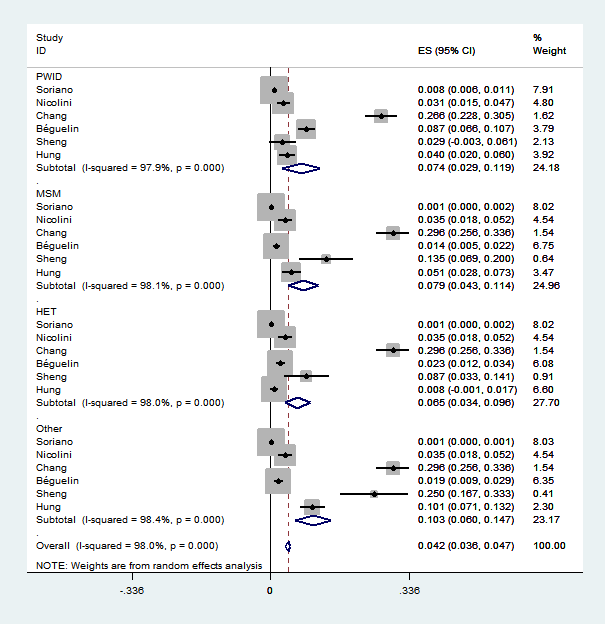

Supplement: Multimedia Appendix 7 [file publichealth_v8i11e37016_app7.png]

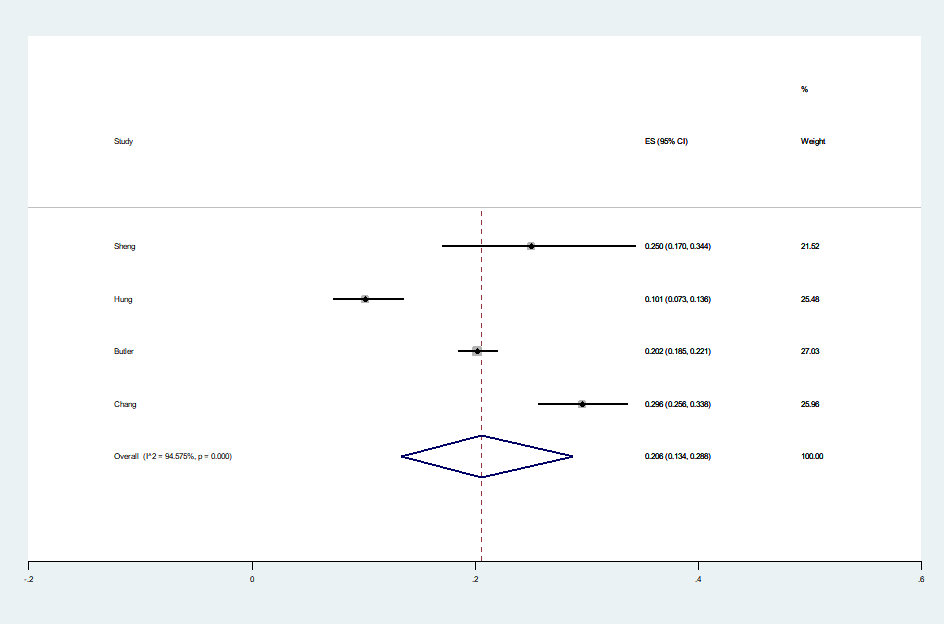

Supplement: Multimedia Appendix 8 [file publichealth_v8i11e37016_app8.png]
